# Editorial

**Published:** 1964-04

**Authors:** 


### EDITORIAL

### CAVING HAZARDS

Within a few miles of Bristol is one of the finest regions in the country for cavers, and there exists in the district a considerable body of experts in caving technique, as well as a well-organized rescue service. But caves, like the wintry moors and the rock faces of our mountains, attract large numbers of the enthusiastic but inexpert, and doctors near the Mendips may be called upon to attend to caving accidents or to consider whether a patient may have exposed himself to risks peculiar to the cave environment. One of these risks, immersion in cold water, was the subject of a paper in our last issue, and its treatment depends very much upon the man on the spot.

But there are other risks that lead to disease only after an interval, and in these cases the connection with a caving expedition may be less apparent. Infections are fortunately rare. British caves are not known to harbour pathogenic fungi, such as *Histoplasma capsulatum;* but water in the Mendip caves comes from a farmed area and may carry the excreta of domestic animals and their more dangerous commensals, rats. At least one infection with *Leptospira* has been known to occur, and other water-borne diseases are possible.

Another danger has been described by R. M. and M. A. M. Williams in the current number of the Transactions of the Cave Research Group (Vol. 6, No. 2). Explosives are sometimes used in caves, and the still atmosphere may not quickly carry away the products of combustion, so that those who return to the site may inhale dangerous gases. Moreover the conditions of working may lead to inadequate tamping of the charge, so that the gases released may not have the expected composition. In particular, nitrogen dioxide may be formed in quantity, and for this gas there is no warning level that the victim will perceive in time to withdraw. In 1960 a caver in South Wales inhaled nitrous fumes after an explosion, and had no symptoms until 13 hours later, when he became breathless, and soon afterwards cyanosed and comatose. He recovered under hospital treatment with oxygen, sedation, diuretics, postural drainage, and suction. His symptoms were due to pulmonary oedema, and it is important to remember that this may develop insidiously and may be fatal without treatment, though if recovery occurs it is complete. Early recognition of the danger, and quick transit to a hospital capable of dealing with respiratory emergencies, may be life-saving.

